# Effects of prenatal exposure to synthetic sex hormones on neurodevelopment: a biological mechanism.

**DOI:** 10.3389/fnmol.2023.1237429

**Published:** 2023-11-09

**Authors:** Marie-Odile Soyer-Gobillard, Laura Gaspari, Françoise Paris, Philippe Courtet, Charles Sultan

**Affiliations:** ^1^Univ Sorbonne, CNRS, Paris, France; ^2^Association HHORAGES-France, Perpignan, France; ^3^Unité d’Endocrinologie-Gynécologie Pédiatrique, Service de Pédiatrie, CHU Montpellier, Univ Montpellier, Montpellier, France; ^4^Centre de Référence Maladies Rares du Développement Génital, Constitutif Sud, Hôpital Lapeyronie, CHU Montpellier, Univ Montpellier, Montpellier, France; ^5^INSERM 1203, Développement Embryonnaire Fertilité Environnement, Univ Montpellier, Montpellier, France; ^6^IGF, Univ. Montpellier, CNRS, INSERM, Montpellier, France; ^7^Department of Emergency Psychiatry and Acute Care, Lapeyronie Hospital, CHU Montpellier, Montpellier, France

**Keywords:** estrogens, progestogens, *in utero* exposure, neurodevelopment, multi-generational impact, biological mechanisms

## Abstract

Since the middle of the 20th century, synthetic sex hormones (estrogens and progestins) have been administered to millions of pregnant or not women worldwide, mainly to avoid miscarriage or for comfort, although their mode of action and their effects on the mother and fetus were ignored. Despite the alerts and the description of somatic and psychiatric disorders in children exposed *in utero*, synthetic estrogens were prohibited for pregnant women only in the 1970s and 1980s, but some progestins are still authorized. In this review, we summarize the psychiatric disorders described in children exposed *in utero* to such hormones, focusing particularly on schizophrenia, bipolar disorders, severe depression, eating disorders, suicide and suicide attempts. Moreover, only in 2017 the mechanism of action of these xenohormones has started to be deciphered. Some studies showed that in the fetus exposed *in utero*, they alter the DNA methylation profile (mainly hypermethylation), and consequently the expression of genes implicated in neurodevelopment and in regulating the sexual organ morphogenesis and also of the promoter of estrogen receptors, located in the amygdala. These deleterious effects may be transmitted also to the next generations, thus affecting the children directly exposed and also the following generations.

## 1. Introduction

Synthetic estrogens, such as diethylstilbestrol (DES) and 17-α-ethinyl estradiol (EE), differ from natural sex hormones in particular by their toxic degradation products. DES, a synthetic non-steroidal diphenol with potent estrogenic activity and very lipophilic, is mainly metabolized through an oxidation reaction to the catechol quinone that forms DNA adducts (Saeed et al., 2009). Such adducts accumulate in the mother’s adipose tissue and may induce modifications in single or double strand DNA during their metabolization (Neault and Tajmir-Riahi, 1996), genotoxic effects in the mother, various hormone-dependent cancers (Newbold, 2008), and deleterious effects in the exposed fetus. The natural estrogenic hormone 17-β-estradiol is a lipophilic compound that should bind to adipose tissue; however, through the detoxifying enzyme cytochrome P-450 in the liver, it is eliminated in the form of water-soluble metabolites in the urine. The synthetic estrogen EE, often associated with DES in formulations, also is lipophilic, and is eliminated through other alternate metabolic pathways that lead to cytochrome P-450 inactivation and inhibition of EE degradation (Wittliff and Andres, 2014). It remains then fixed in maternal adipose tissue before a future fetus contamination during the next pregnancy. In 1980, EE was banned by the European Community for pregnant women, but it is still used in oral contraceptives worldwide (Soyer-Gobillard et al., 2021b). Tournaire et al. (2014) retraced the history of DES that was initially considered a miracle drug to reduce pregnancy complications and was finally withdrew from the market due to the many complications (particularly clear cell carcinoma) observed in people exposed *in utero* (Herbst et al., 1971). However, the authors did not discuss psychiatric disorders in the offspring of women who took DES or EE. From the 1950s to the late 1970s, synthetic estrogens were prescribed to millions of women in the world during pregnancy to prevent miscarriage and premature birth, to suppress lactation after childbirth, or to treat infertility and other gynecological disorders. Although various warnings were published as early as 1938 by Lacassagne (1938), shortly after DES synthesis by Dodds et al. (1938), evidence has been accumulating that DES and EE lead not only to somatic effects in adulthood, but also to neurodevelopment alterations in the exposed fetus that may increase the risk of psychiatric disorders. Indeed, behavioral disorders have been described in young adults who were exposed *in utero* to EE or DES: depression (Pillard et al., 1993) and anxiety (Katz et al., 1987; Saunders, 1988; Verdoux, 2000), schizophrenia (Katz et al., 1987), anorexia and bulimia (Geary, 1998). However, studies on DES and EE neuropsychiatric effects are scarce. Kebir and Krebs (2012) identified 10 relevant epidemiological studies, including the works by Vessey et al. (1983), Verdoux et al. (2007), and O’Reilly et al. (2010). Vessey et al. (1983), through a mail survey in a sample from a double-blind study performed in 1953 (650 women treated with DES and 650 with placebo), were the first to describe an increase in psychiatric disorders (mainly depression and anxiety) in the group that was exposed *in utero* to DES compared with the untreated group. Verdoux et al. (2007) analyzed information provided by 1,352 women who received DES during pregnancy (1,680 *in utero* exposed children) and concluded that exposure to DES was not linked to suicide, psychiatric consultations or psychiatric hospitalizations. O’Reilly et al. (2010) analyzed data from the Nurses’ Health Study II (76,240 American women among whom 1,612 reported prenatal DES exposure) and showed that the risk of depression was increased in the exposed group. Kebir and Krebs (2012) underlined the limitations of these epidemiological studies. Besides depression and anxiety, these studies did not evaluate other psychiatric disorders. Moreover, the mechanisms of action linking DES and psychiatric diseases was not known. Fewer works have been published on EE effects. Caston’s group (Dugard et al., 2001; Arabo et al., 2005) showed that EE injection in pregnant rats (doses equivalent to those used in humans: 15 g/kg-1 per day versus 19 g/kg-1 per day) increased the abortion rate and induced anxiety-like and depression-like behaviors in the offspring. Moreover, Sandner et al. (2004) observed histological alterations in the anterior part of the hippocampus in young rats after *in utero* exposure to EE. The hippocampus expresses many estrogen receptors in the prenatal period. In 2008, Ogiue-Ikeda et al. (2008) reported that estrogens (i.e., DES) and other endocrine disruptors (i.e., bisphenol A) can disturb synaptic plasticity in the hippocampal subregions CA1 and CA3, and modulate long term depression and spinogenesis. Their regulatory mechanism remains unclear. In 2020, Sumi and Harada (2020) presented a tentative model to explain the reproducing induction of neuronal plasticity, long term potentiation and long term depression. This complex mechanism implicates a competition between exocytosis and endocytosis of AMPA receptors (alpha-amino-3-hydroxy-5-methyl-isoxasolepropionic acid receptors, the ionotropic transmembrane receptors for glutamate) that mediate synaptic transmission in the central nervous system (CNS) at the level of presynaptic membrane. Bencsik et al. (2023) showed that AMPA receptor trafficking plays a major role in the functioning of neurons and neural networks. Briefly, data have been accumulating suggesting that synthetic sex hormones negatively affect the neurodevelopment of the exposed fetus.

## 2. DES and EE effects in children exposed *in utero*: several types of psychiatric disorders

Xenoestrogens are considered as study models for other endocrine disruptors, and their effects on the CNS have been investigated in the last decades. Two previously discussed epidemiological studies (Vessey et al., 1983; O’Reilly et al., 2010) found higher frequency of depression and anxiety in participants exposed *in utero* to DES than in untreated controls. Moreover, analysis of data from a French national survey carried out by the DES-France Network Association (2,566 women exposed to DES *in utero* and 2,967 unexposed women) highlighted that women exposed *in utero* were 1.7 times more likely to consult a psychiatrist, suggesting that they are at higher risk for psychiatric disorders (Verdoux et al., 2017).

Soyer-Gobillard et al. (2016) analyzed questionnaire data from 529 families [*n* = 1,182 children among whom 740 were (−20 who were stillborn) exposed *in utero* and 262 post-exposed and 180 were unexposed as intra-familial control] that are part of the HHORAGES-France Association (Halt to Artificial HORmones for Pregnancy). This association was created in 2002 and includes families (currently *n* = 1,350) of women who took synthetic sex hormones during pregnancies and their children and grandchildren. The authors found severe psychotic disorders, such as schizophrenia (22.9%), behavioral disorders (15.1%), depression (34.4%), eating disorders (11.3%), suicide (4.4%) and attempted suicide (85%), in post-adolescence and adulthood compared with the general population (Table 1).

**Table 1:** Reprinted with permission from Prevalence of psychiatric disorders and comparison with the general population in a population of 982 (1002–20 stillborn babies) DES-exposed and post-DES children. Courtesy of *Gynecological Endocrinology* (Taylor & Francis) (Soyer-Gobillard et al., 2016).

	Group 2 Des-exposed (*n* = 740–20)	Group 3 Post-DES (*n* = 262)	Group 1 Firstborn pre-DES (*n* = 180)	General population
Behavioral disorders	(*n* = 109) (15.1%)	(*n* = 1) (0.4%)	(0%)	(3%)
Eating disorders	(*n* = 81) (11.3%)	(*n* = 2) (0.8%)	(0%)	(1.6%)
Schizophrenia	(*n* = 165) (22.9%)	(*n* = 6) (2.3%)	(0%)	(1%)
Depression Suicides	(*n* = 248) (34.4%)	(*n* = 9) (3.4%)	(0%)	(6.3%)
Attempts	(*n* = 612) (85%)	(*n* = 30) (11.5%)	(0%)	(0.3%)
Death	(*n* = 32) (4.4%)	(*n* = 1) (0.4%)	(0%)	(0.02%)

### 2.1. Synthetic sex hormones affect the epigenetic profile during neurodevelopment

From 2007, the partnership between the HHORAGES Association and the Centre d’Etudes et de Recherche Clinique (CERC) of Sainte Anne Hospital, Paris, France, resulted in the first molecular study showing a link between exposure to synthetic estrogens and psychiatric disorders (Rivollier et al., 2017). The authors analyzed Differentially Methylated Regions (DMR) in genes involved in neurodevelopment and implicated in psychiatric vulnerability in 69 siblings from 30 HHORAGES families among whom at least one child was prenatally exposed to DES. A neuropsychiatric evaluation was performed and peripheral whole blood samples were collected to assess methylation variations in 411,947 CpG islands in exposed (*n* = 37) and unexposed (*n* = 32) individuals. Psychiatric disorders were observed in both groups, but their rate was higher in the exposed group: psychosis (*n* = 4 schizophrenia and *n* = 3 schizoaffective disorder in the exposed group and none in the unexposed group), bipolar disorders, depression, anxiety disorders. Specific differentially (hyper) methylated regions were identified in *ZFP57* and in a region close to the *ADAM TS9* promoter (hypermethylated in the exposed group versus unexposed siblings). *ZFP57* hypermethylation was observed in exposed children with psychiatric disorders. This gene is expressed very early during development, and regulates the transcription of many genes implicated in CNS development (Quenneville et al., 2011). *ADAM TS9*, a disintegrin and metalloproteinase, also is involved in CNS development and more precisely in synaptic plasticity, neurorepair, angiogenesis and inflammation mechanisms (Lemarchant et al., 2013) and in regulating organ morphology, particularly uterus and reproductive system (Mittaz et al., 2004), which are often altered in DES children. Interestingly, in a cohort of adolescents also followed at Sainte Anne Hospital, different DNA methylation alterations were observed in participants who converted or not to psychosis (Kebir et al., 2017). These alterations consisted of global hypermethylation of the genome in these young people not exposed to DES but who became schizophrenic during this study, while specific genes were concerned in children exposed to DES from the HHORAGES cohort. Conversely, Harlid et al. (2015) did not detect any DNA methylation difference between 100 women who reported *in utero* exposure to DES and 100 unexposed women (both between 40 and 59 years of age), but they did not analyze DMRs.

### 2.2. Multigenerational impact of synthetic estrogens in the third and fourth generations

**Figure 1:**
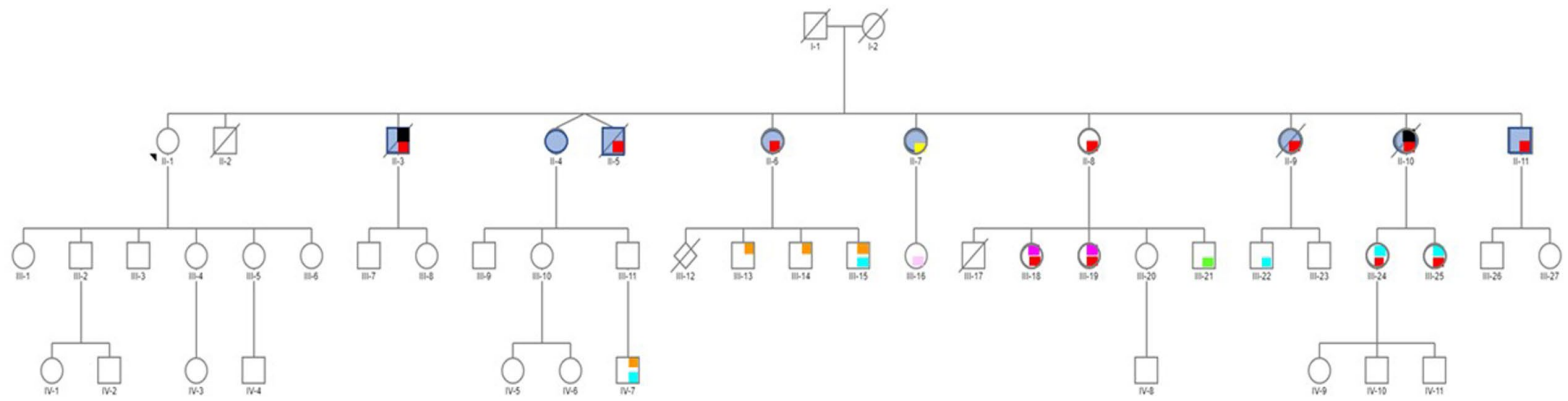
Pedigree of an informative family whose mother (I-2) was treated with DES (30 mg/day) for 3 months after each delivery to inhibit lactation. Only the first child (II-1) was not exposed to DES in utero. Daughter II-1 and her descendants do not have any psychiatric disorder. No history of psychiatric disorders was reported for the maternal and paternal sides. Legend: red = bipolar disorder, yellow = psychosis borderline, blue = attempted suicide(s), black = suicide, orange = autism spectrum disorder (ASD), light blue = dyspraxia and learning disabilities, light pink = mood and sleep disorders, green = behavioral disorders, fuchsia = eating disorders. Reprinted from Prenatal exposure to diethylstilbestrol and multigenerational psychiatric disorders: an informative family by Soyer-Gobillard et al. (2021b, licensed under CC-BY 4.0).

Studies on the effect of synthetic estrogens on the neurodevelopment in the children of individuals exposed *in utero* (i.e., third generation) are still rare. In an American study on 47,450 participants enrolled in the “Nurses’ Health Study II” (the participants, their mothers, and their live-born children), Kioumourtzoglou et al. (2018) concluded that exposure to DES was associated with higher risk of attention deficit/hyperactivity disorder (ADHD) in grandchildren. More recently, in a case report, Soyer-Gobillard et al. (2021a, 2022) suggested a multigenerational and perhaps transgenerational DES effect on neurological development, bipolar disorders and Autism Spectrum Disorders (ASD) in grandchildren and a great-grandson. In this family of 11 children, the mother took DES for 3 months after each birth to stop lactation. Therefore, only the oldest daughter was not exposed, and was used as intra-familial control. This daughter and her descendants did not report any psychiatric/somatic disorder. Conversely, the exposed siblings (second generation) reported psychiatric problems (bipolar disorders, eating disorders, suicide behaviors), generally associated with endometriosis in girls and hypospadias in boys. Moreover, 10 of the 19 surviving DES-exposed grandchildren (third generation) reported ASD (boys), bipolar disorder (girls), dyspraxia and learning disabilities, mood and behavioral disorders, and eating disorders, often associated with endometriosis (Gaspari et al., 2021) and hypospadias, as shown in Soyer-Gobillard et al. (2021a) (Figure 1 and Table 1). Lastly, one of the seven DES-exposed great-grandchildren (fourth generation; aged from 0 to 18 years) presented dyspraxia and ASD. The other six great-grandchildren are still very young. This case study suggest that DES may have an effect over several generations.

## 3. Synthetic progestogens (progestins)

Steroid hormones contribute to regulate the reproductive function. Progestins (bioidentical and non-bioidentical forms) were administered to pregnant women but are now mostly banned (Table 2), except (in France) for 17-α-hydroxyprogesterone caproate (often used as delayed-release progestin). This synthetic hormone is prescribed to pregnant women (banned in 2000 and reauthorized in 2011 in France) to prevent repeated abortions due to luteal insufficiency, and in case of risk of premature delivery.

**Table 2:** Bioidentical (B) and not bioidentical (NB) progestins currently prescribed to women or withdrawn from the market (but used in the past).

(B)^a^ and NB progestins prescribed for contraception or for hormone replacement therapy (HRT) women	Progestins prescribed to 62 pregnant women. (HHORAGES data)^b^	Date of withdrawal for pregnant
Medroxyprogesterone acetate: B: HRT, contraception	Chlormadinone acetate (11)	1970
Derivatives:	17-α-hydroxyprogesterone heptanoate (13)	2002
17-α-hydroxyprogesterone heptanoate, NB: HRT, contraception 17-α-hydroxyprogesterone caproate, NB: HRT, contraception	17-α-hydroxyprogesterone caproate (32) (often used as progestin delay)	2000 Reauthorized 2011
Dydrogesterone, NB: HRT, contraception	Dydrogesterone (4)	Contraindication
Micronized progesterone, B: HRT, contraception	Micronized progesterone (4)	Contraindication
Norgestrel, levonorgestrel^c^, NB: HRT, contraception	Levonorgestrel (1)	Contraindication
Norethisterone, norethindrone acetate, NB: HRT, contraception	Norethisterone base (1)	Contraindication

Note that 17-α-hydroxyprogesterone, which has been associated with psychiatric disorders (*n*=32 individuals exposed in utero) in the HHORAGES cohort, was prohibited for pregnant women in 2000, but was reauthorized in 2011. Moreover, other progestins that were contraindicated or prohibited for pregnant women are authorized in contraception. Courtesy of Academic Press/Elsevier. Reproduced from Soyer-Gobillard et al. (2021c).

Reproduced from Soyer-Gobillard et al. (2016), courtesy by Gynecological Endocrinology.

Micronized progesterone is considered as B, derived from diosgenin in soybean (an endocrine disruptor) or inedible wild yam. From M. O. Soyer-Gobillard.

a Pattimakiel and Thacker (2011).

b Based on doctors’ prescription data.

c Zou et al. (2017), Li et al. (2018). HHORAGES data.

Like natural progesterone, progestins activate the two progesterone receptor (PR) isoforms: PR-A and PR-B. Cartwright et al. wrote that “Both isoforms can regulate transcription by the classical mechanism of either enhancing gene expression via the direct binding to progesterone response elements in the promoter region of specific target genes or by repressing gene expression through tethering to DNA-bound transcription factors” (Cartwright et al., 2023). Moreover, the authors suggested that progestin effects *via* PR-A are influenced by the receptor density.

### 3.1. Progestin effects in individuals exposed *in utero* (second generation)

The analysis by liquid chromatography-mass spectrometry of frozen amniotic fluid samples (Baron-Cohen et al., 2015) highlighted higher sex hormone concentrations (especially progesterone and 17-α-hydroxyprogesterone) in 128 boys with ASD than in matched controls (Danish cohort). This was the first study showing higher fetal steroidogenic activity in ASD. Later, these authors (Baron-Cohen et al., 2020) noted in the same cohort that the concentrations of estrogen and progesterone were 50% higher in the amniotic fluid samples of the boys with ASD compared with the matched controls.

Paul Yao’s group (Zou et al., 2017) observed autism-like behavior in the offspring of rats that received progestin (levonorgestrel, LNG) with/without EE (but not to EE alone) from day 1 to day 21 of pregnancy. They found that at the age of 10 weeks, the estrogen receptor (ER)β gene promoter was hypermethylated in the offspring of rats treated with LNG with/without EE, leading to downregulation of ERβ and its target genes in brain. In a case–control epidemiological study in Hainan province (an island of 8 million inhabitants in China), the same group (Li et al., 2018) reported higher risk of ASD after *in utero* exposure to progestins. Among 37,863 children aged 0–6 years, they selected 235 children with ASD and 682 age- and sex-matched controls. By comparing the available data on their mothers’ medical history they found that use of progestin to prevent abortion and of progestin contraceptives at conception time, and surprisingly, also consumption of progestin-contaminated seafood in the first three months of pregnancy were associated with ASD. The authors wrote that in China, seafood farmers frequently use estrogen-progestogen contraceptives to prevent egg laying and to obtain bigger and fatter fish and shrimp more quickly. This may contribute to explain the increasing incidence of ASD in China (and in other countries).

Our group (Soyer-Gobillard et al., 2019a) identified 46 families (*n* = 115 children) in the HHORAGES cohort exposed to progestins alone, especially 32 children *in utero* exposed to the reauthorized 17-α-hydroxyprogesterone caproate (Table 2). Schizophrenia, bipolar disorders, severe depression, behavioral disorders, aggressiveness, and eating disorders were reported by the *in utero* exposed children. Comparison with data on 1,002 individuals *in utero* exposed to synthetic estrogens from the HHORAGES cohort (Soyer-Gobillard et al., 2016) showed minor differences for boys. However, bipolar disorders seemed relatively more frequent in boys exposed to progestins than to synthetic estrogens, whereas behavioral disorders were more frequent in the latter group. In girls, results were comparable between individuals exposed to progestins and to synthetic estrogens. Whatever the exposure type (progestins or synthetic estrogens), bipolar disorders and severe depression were most frequently reported by girls and schizophrenia by boys (Soyer-Gobillard et al., 2019b).

### 3.2. A complex mechanism: ERβ Receptor and GABAergic system implication In progestin deleterious effects

Steroids that have an effect on the nervous system, such as progesterone, contribute to shaping the CNS structure and function (e.g., neurogenesis and glial cell and synapsis plasticity). Progesterone also modulates the development of different neuron types and myelinization and also the activity of several signaling pathways that have been implicated in the pathophysiology of psychosis, particularly the dopaminergic, glutamatergic, and GABAergic systems. Moreover, dihydroprogesterone and allopregnanolone (or iso-pregnanolone), two progesterone metabolites (Schumacher et al., 2014), are potent ligands of the GABA-A receptor. GABA receptor activity/neural activation upon progestin exposure during pregnancy may alter GABAergic signaling. This signaling may be altered throughout cell proliferation, migration, maturation, and integration into cortical circuits. Modifications of these factors could contribute to the development of psychiatric disorders (e.g., schizophrenia, depression, ASD) later in life (Schmidt and Mirnics, 2015). Natural progesterone acts by modulating gene transcription after binding to progesterone nuclear receptors, and by activating signal transduction pathways. The mechanisms underlying the development of psychiatric disorders/ASD in later life following *in utero* exposure to progestins could be complex and have not been fully elucidated yet. On the other hand, the link between *in utero* exposure to progestin and the appearance of such disorders in the offspring seems obvious. The hypermethylation of the estrogen and progestogen receptor promoters upon *in utero* exposure to LNG (Zou et al., 2017) suggests that the molecular machinery and its effects on offspring might be the same as with synthetic estrogens, although no study on progestin effects on the third generation has been published yet. Only recently Skosana et al. (2019), showed for the first time that progestins are very rapidly degraded (less than 24 h) in mammalian cell lines and are significantly metabolized in human cervical tissue. This could explain why the toxic effect of these xenohormones is more quickly harmful.

## 4. Discussion and conclusion: other avenues to explore

Synthetic estrogens act by modulating the transcription of target genes (Coons and Korach, 2023), as well as progestins (Cartwright et al., 2023). Previous studies presented in this review suggest that genes expressed in the hippocampus and/or amygdala and implicated in ASD (amygdala) and psychosis (both brain regions) may be affected by *in utero* exposure to progestins and/or synthetic estrogens. Several studies based on observations over several generations suggest the transmission of psychiatric/somatic disorders to the offspring of women treated with synthetic hormones (mainly DES): ADHD in a large population in the USA (Kioumourtzoglou et al., 2018) and ASD, bipolar disorders and ADHD in an informative family (Soyer-Gobillard et al., 2021c), often associated with somatic morbidities (Gaspari et al., 2021; Zamora-Leon, 2021). In all cases, hypermethylation of specific genes has been implicated. Synthetic estrogens, progestins, and their combination may trigger similar psychiatric disorders in *in utero* exposed children and also somatic disorders. Due to the widespread use of synthetic hormones in oral contraceptives, more studies are urgently needed to precisely determine their epigenetic effects (Strifert, 2015). In this review we focused on xenohormones administered during pregnancy that may induce deleterious epigenetic changes (hypermethylation) in genes implicated in neural development. This risk could be extrapolated to other environmental contaminants during pregnancy. The current state of knowledge has only allowed us to propose the basis of the mechanisms of action of sex xenohormones on brain development in children exposed *in utero*. However, other mechanisms should also be investigated, particularly non-coding microRNAs, histone modifications, and extracellular vesicles (Montjean et al., 2022). Epidemiological surveys should also be carried out in children and grandchildren of women who used progestins, associated or not with synthetic estrogens.

Focus on key facts:

- We analyzed the effects on CNS of *in utero* exposure to synthetic hormones (estrogens and progestins) and their mechanism of action.
- Xenoestrogens may induce psychosis and other psychiatric disorders by altering the DNA methylation profile (increase) of genes implicated in neurodevelopment.
- Similarly, progestins may contribute to ASD development by altering the DNA methylation profile (increase) of the ER β receptor gene promoters.
- Two brain regions, hippocampus and amygdala, are specifically at risk in children exposed *in utero* to progestins or/and estrogens through alteration of GABAergic signaling and ER β receptor gene expression.
- This review also highlighted multigenerational and possibly transgenerational effects of synthetic estrogens on the third and fourth generations (both not exposed *in utero*).

## Author’s note

Association HHORAGES-France, a patient association, is registered at the Epidemiological Portal of French Health INSERM (French National Institute for MedicalResearch) and AVIESAN (National Alliance for Life Sciences and Health) (epidemiologiefrance.aviesan.fr). (CNIL N°1006460).

## Author contributions

M-OS-G drafting the manuscript. LG, FP, PC, and CS: revising the manuscript critically for intellectual content. All authors contributed to the article and approved the submitted version.
